# USP22 Is Useful as a Novel Molecular Marker for Predicting Disease Progression and Patient Prognosis of Oral Squamous Cell Carcinoma

**DOI:** 10.1371/journal.pone.0042540

**Published:** 2012-08-03

**Authors:** Songlin Piao, Yanlong Liu, Jing Hu, Fulin Guo, Jie Ma, Yao Sun, Bin Zhang

**Affiliations:** 1 Department of Oral and Maxillofacial Surgery, The First Affiliated Hospital of Harbin Medical University, Harbin, Heilongjiang, People's Republic of China; 2 Department of Colorectal Surgery, The Third Affiliated Hospital of Harbin Medical University, Harbin, Heilongjiang, People's Republic of China; 3 Department of Breast Medical Oncology, The Third Affiliated Hospital of Harbin Medical University, Harbin, Heilongjiang, People's Republic of China; 4 Hard Tissue Lab, The Second Affiliated Hospital of Harbin Medical University, Harbin, Heilongjiang, People's Republic of China; 5 Department of Oral and Maxillofacial Surgery, The Second Affiliated Hospital of Harbin Medical University, Harbin, Heilongjiang, People's Republic of China; Okayama University, Japan

## Abstract

**Background and Objective:**

The significance of ubiquitin-specific protease 22 (USP22) as a potential marker has been growing in the field of oncology. The aim of this study was to investigate the role of USP22 and the association with its potential targets in oral squamous cell carcinoma (OSCC).

**Methods:**

Immunohistochemistry was used to determine the expression of USP22 protein in 319 OSCC patients in comparison with 42 healthy controls. The clinical correlations and prognostic significance of the aberrantly expressed protein was evaluated to identify novel biomarker of OSCC.

**Results:**

The incidence of positive USP22 expression was 63.32% in 319 conventional OSCC tissues. The protein expression level of USP22 was concomitantly up-regulated from non-cancerous mucosa to primary carcinoma and from carcinomas to lymph node metastasis (*P*<0.001). Moreover, statistical analysis showed that positive USP22 expression was positively related to lymph node metastasis, Ki67, Cox-2 and recurrence. Furthermore, it was shown that patients with positive USP22 expression had significantly poorer outcome compared with patients with negative expression of USP22 for patients with positive lymph nodes. Multivariate Cox regression analysis revealed that USP22 expression level was an independent prognostic factor for both overall survival and disease-free survival (*P*<0.001 and *P*<0.001, respectively). Cancer cells with reduced USP22 expression exhibited reduced proliferation and colony formation evaluated by MTT and soft agar assays.

**Conclusion:**

To our knowledge, this is the first study that determines the relationship between USP22 expression and prognosis in OSCC. We found that increased expression of USP22 is associated with poor prognosis in OSCC. USP22 may represent a novel and useful prognostic marker for OSCC.

## Introduction

Deubiquitinating enzymes regulate a number of cellular mechanisms including pre-implantation, growth and differentiation, oncogenesis, cell cycle progression, transcriptional activation, and signal transduction [1]. Recently, Lee et al have identified a novel human deubiquitinating enzyme gene, USP22, is ubiquitously expressed in various adult tissues and at the early embryonic stages [2]. Increasing evidence places USP22 at the core of a multitude of physiological and pathological processes. First, USP22 is a part of Polycomb/cancer stem cell signature which uniformly exhibit a marked propensity toward metastatic dissemination as well as a therapy resistance phenotype [3]. Second, USP22 comprises a second enzymatic subunit of hSAGA, which is required for activatordriven transcription [4]. Third, USP22 plays a direct functional role in regulating cell-cycle progression [5]. Thus, McMahon et al propose that the link between the metastasis marker USP22, hSAGA-dependent transcription, and cell-cycle progression suggests the possibility that loci regulated by USP22 may encode attractive therapeutic targets [5].

More recently, Pijnappel et al propose that targeted deubiquitylation of histones is intimately linked to transcription activation, epigenetic regulation, and cancer progression [6]. Within SAGA, USP22 deubiquitylates histone H2B and H2A in vitro [5], [7]. We proposed that USP22 may be an important regulator of biological processes and potential targets for the treatment of a variety of human diseases, including several types of malignancies. In fact, USP22 is one of a small set of marker genes capable of predicting metastatic potential and therapeutic outcome in human cancer such as colorectal cancer [8], [9], breast cancer [10], and bladder cancer [11]. However, clinicopathologic significance of USP22 in oral squamous cell carcinoma (OSCC) has not yet been elucidated. In this study, we examined firstly the protein expression of USP22 in human OSCC tissues by immunohistochemical staining. Furthermore, the correlation between USP22 level and clinicopathologic features, including patients outcome, was analyzed. Our study will be attributed to exploit for both molecular diagnostics and the development of novel strategies to cancer.

## Materials and Methods

### Patients and tumor samples

The 403 formalin-fixed paraffin embedded specimens used to Immunohistochemistry (IHC) were collected from 319 OSCC patients undergoing surgery between January 2004 and December 2005, and were grouped as non-cancerous mucosa (n = 42) and primary carcinoma (n = 319) and lymph nodal metastasis (n = 42). The matching normal and lymph nodal metastasis was available for 42 of the primary carcinomas. Data were retrieved from patients' operative and pathological reports, and follow-up data were obtained by phone, letter, and the outpatient clinical database. This study comprised 231 men and 88 women aged from 24 to 92 years old, mean age was 58.8 years. Primary cancers were evaluated in accordance with the American Joint Committee on Cancer, 7th ed., staging system. All patients were followed-up to August 2011 or until death. Median follow-up time for survivors was 41 months (range 3.90–87.10 months). No patient received preoperative chemotherapy or radiotherapy. According to clinical requirement, Ki67 and Cox-2 are also routinely stained for all OSCC patients.

The written informed consent had been obtained from all the patients, and this study was approved by the Ethical Committee of the First and Second Affiliated Hospital of Harbin Medical University. The study was retrospective.

### Immunohistochemistry

Immunophenotype analysis of USP22 was done as previously described [8], [9]. In brief, the formalin-fixed, paraffin-embedded sections (4 µm) were deparaffinized in xylene and rehydrated in a graded series of ethanol solutions. The sections were subsequently submerged in EDTA (pH 8) and autoclave at 121°C for 5 min to retrieve the antigenicity. Endogenous peroxidase was quenched with 3% H_2_O_2_ for 15 min. After washing with PBS, the sections were incubated with USP22 antibody (Abcam, ab4812, diluted at 1∶200) overnight at 4°C. The sections were incubated with peroxidase-conjugated streptavidin for 30 min and the reaction products were visualized with diaminobenzidine as a chromogen and counterstained with commercial hematoxylin. The percentage of positive cells was determined by counting 500 cells in five random areas per section. Nuclear immunostaining results for USP22 were evaluated using the semiquantitative assessment [12], which calculated the staining intensity and the percentage of positive cells. IHC staining was scored according to the following criteria:−, none of the cells stained; +, 1–20% of the cells stained; ++; 20–50% of the cells stained; +++, 50–100% of the cells stained. IHC score of USP22 expression was [0 (negative)≤score <2+] and [2+ ≤score <3+], which represented negative and positive expression, respectively.

### Cell culture, siRNA transfection and Western blotting analysis

The human OSCC cell lines Tca-8113 and Cal-27 were purchased from cell bank of the Chinese Academy of Sciences in Shanghai. The two cell lines were cultured at 37°C in 5% CO_2_ atmosphere in DMEM medium (Hyclone, Logan, UT), supplemented with 10% bovine calf serum (Hyclone). For the RNAi-down regulation of USP22, the USP22 sequence specific siRNA and negative control siRNA were designed and synthesized by Invitrogen (Carlsbad, CA) as described previously [13]. Total cell lysates from the cultured cell were prepared and the protein concentrations were determined with the method described by Liu [13].

### Cell viability and colony formation

Cell viability of Tca-8113 and Cal-27 cells treated with USP22 siRNA was determined as we previously described [14]. Colony formation of Tca-8113 and Cal-27 cells incubated in the presence of USP22 siRNA was evaluated as described [15]. Data points represent mean±SD in one experiment repeated at least twice.

### Statistical analysis

All statistical analyses were performed by using the SPSS software version 17.0. Differences were considered statistically significant when *P* values were <0.05. The chi-square test was used to compare USP22 expression differences between different groups. The association between the USP22 protein and clinicopathologic parameters was tested using the chi-square and Fisher's exact tests. For analysis of the follow-up data, overall survival (OS) and disease-free survival (DFS) were evaluated using the Kaplan-Meier method and log-rank tests. Cox proportional hazards regression analysis was performed to assess the association of USP22 expression with risk of recurrence and death using OS and DFS, respectively.

## Results

### Elevated expression of USP22 protein in OSCC

**Figure 1:**
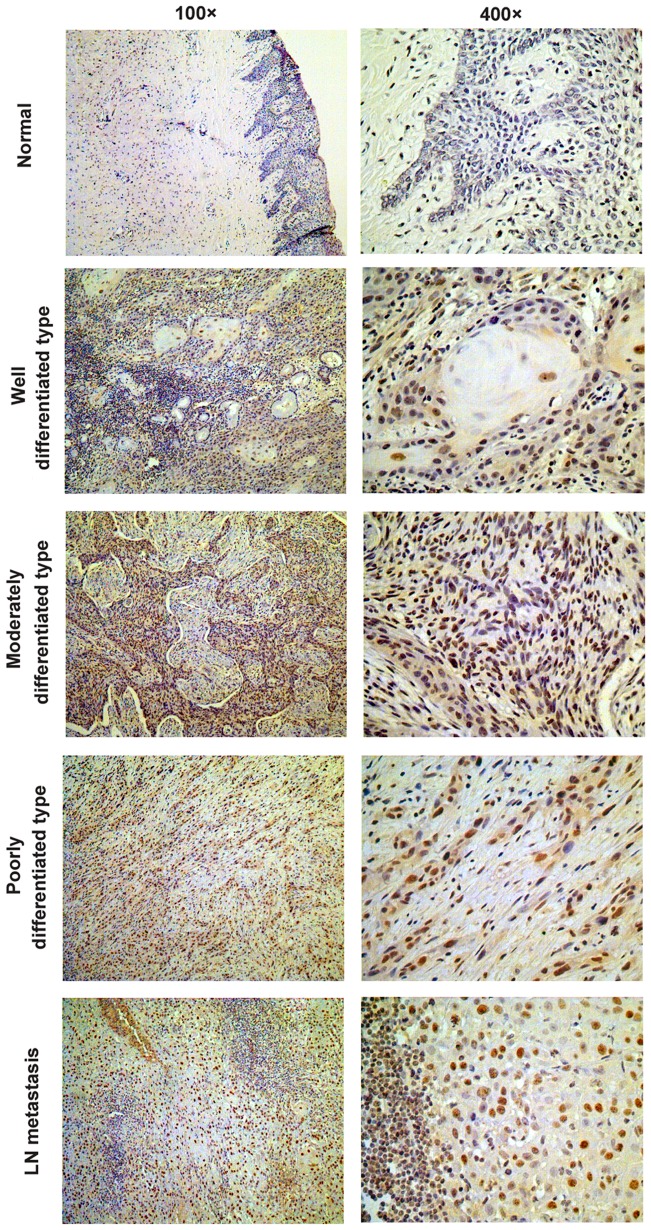
Immunohistochemistry for USP22 in the specimens. Positive USP22 staining in OSCC tissues appeared as brown particles which were mainly localized within the nuclei, with minute staining in the cytoplasmic and cell membrane as well as some scattered infiltrated lymphocytes.

**Figure 2:**
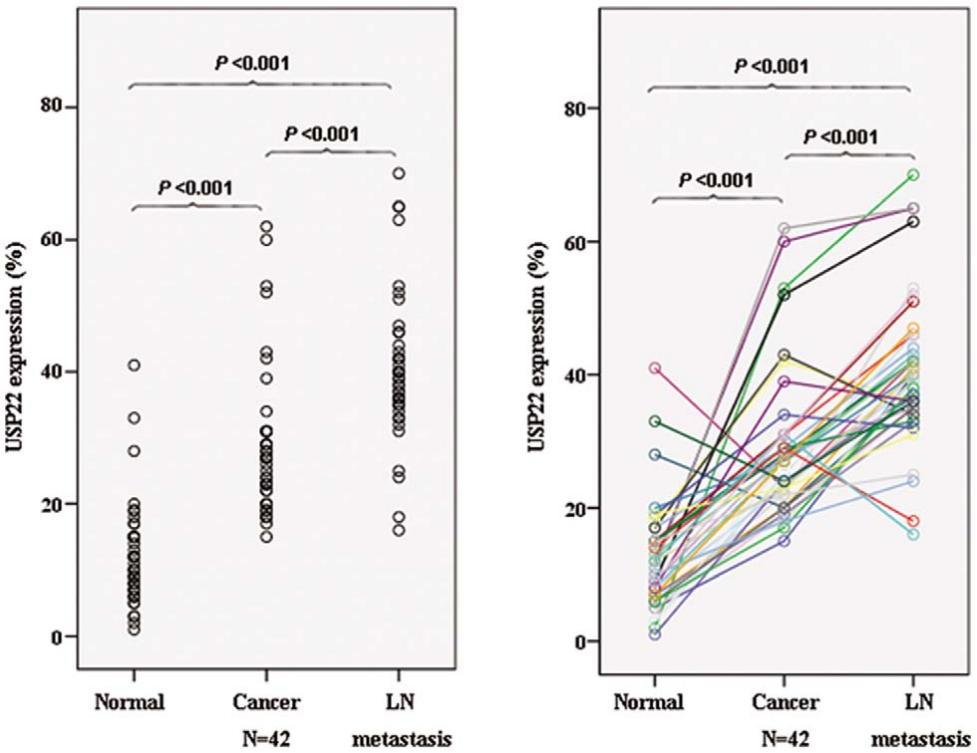
Increase of USP22 staining in OSCC progression. The protein expression level of USP22 was concomitantly up-regulated from non-cancerous mucosa to primary carcinoma and from carcinomas to lymph node metastasis (*P*<0.001).

In agreement with the proposed oncogenic role of the USP22, we evaluated the protein expression level of USP22 in primary OSCCs (n = 319), the paired non-cancerous mucosa tissues (n = 42), and lymph node metastasis tissues (n = 42). We found high USP22 staining in OSCC tissues appeared as brown particles which were mainly localized within the nuclei, with minute staining in the cytoplasmic and cell membrane as well as some scattered infiltrated lymphocytes (Figure 1). The USP22 expression increased significantly from non-cancerous mucosa to carcinomas (*P*<0.001) and from carcinomas to lymph node metastasis (*P*<0.001) (Figure 2). Among the 319 OSCC samples, the positive frequency was 63.32% for USP22.

### Correlation of USP22 with clinicopathologic findings

The correlation between the protein expression of USP22 and clinicopathologic variables of OSCC were shown in Table 1. Positive expression of USP22 were significantly correlated with primary tumor site (χ^2^ = 51.321, *P*<0.001), tumor size (χ^2^ = 15.868, *P*<0.001), differentiation (χ^2^ = 50.029, *P*<0.001), T classification (χ^2^ = 77.196, *P*<0.001), N classification(χ^2^ = 44.599, *P*<0.001), M classification (χ^2^ = 21.743, *P*<0.001), AJCC stage (χ^2^ = 108.994, *P*<0.001), Ki-67 status (χ^2^ = 24.685, *P*<0.001), and COX-2 status (χ^2^ = 17.174, *P*<0.001).

**Table 1:** Correlation between USP22 protein expression level and clinicopathological variables.

Characteristcs	No.(%)	USP22 Expression		κ^2^, *P*
		Negative, n (%)	Positive, n (%)	
**Patient included**	319 (100)	117 (100)	202 (100)	
**Gender**				0.702, 0.402
Male	231(72.4)	81(69.2)	150(74.3)	
Female	88(27.6)	36(30.8)	52(25.7	
**Age(years,mean,range)**	58.8(24–92)			0.362, 0.548
≤50	131(41.1)	45(38.5)	86(42.6)	
>50	188(58.9)	72(61.5)	116(57.4)	
**Primary tumor site**				51.321, <0.001
Buccal	67(21.0)	47(40.2)	20(9.9)	
Tongue	144(45.1)	52(44.4)	92(45.5)	
Gingival	57(17.9)	8(6.8)	49(24.3)	
Other	51(16.0)	10(8.5)	41(20.3)	
**Tumor size (cm)**				15.868, <0.001
<3	143(44.8)	70(59.8)	73(36.1)	
≥3	176(55.2)	47(40.2)	129(63.9)	
**Differentiation**				50.029, <0.001
Well/moderate	212(66.5)	107(91.5)	105(52.0)	
Poor	107(33.5)	10(8.5)	97(48.0)	
**pT classification** a				77.196, <0.001
T1/2	106(33.2)	75(64.1)	31(15.3)	
T3/4	213(66.8)	42(35.9)	171(84.7)	
**pN classification** b				44.599, <0.001
N0	124(38.9)	74(63.2)	50(24.8)	
N1–3	195(61.1)	43(36.8)	152(75.2)	
**pM classification** c				21.743, <0.001
M0	266(83.4)	113(96.6)	153(75.7)	
M1	53(16.6)	4(3.4)	49(24.3)	
**AJCC stage** d				108.994, <0.001
I	54(16.9)	49(41.9)	5(2.5)	
II	77(24.1)	38(32.5)	39(19.3)	
III	125(39.2)	23(19.7)	102(50.5)	
IV	63(19.7)	7(6.0)	56(27.7)	
**Ki- 67 status**				24.685,<0.001
0–25%	124(38.9)	64(54.7)	60(29.7)	
26%–50%	66(20.7)	25(21.4)	41(20.3)	
51%–75%	67(21.0)	16(13.7)	51(25.2)	
76%–100%	62(19.4)	12(10.3)	50(24.8)	
**COX-2 status**				17.174,<0.001
Negative	118(37.0)	61(52.1)	57(28.2)	
Positive	201(63.0)	56(47.9)	145(71.8)	

a : pT represents primary tumor;

b : pN represents regional lymph nodes;

c : pM represents distant Metastasis;

d : AJCC represents American Joint Committee on Cancer.

### Analysis of correlation of USP22 with therapy outcome

**Figure 3:**
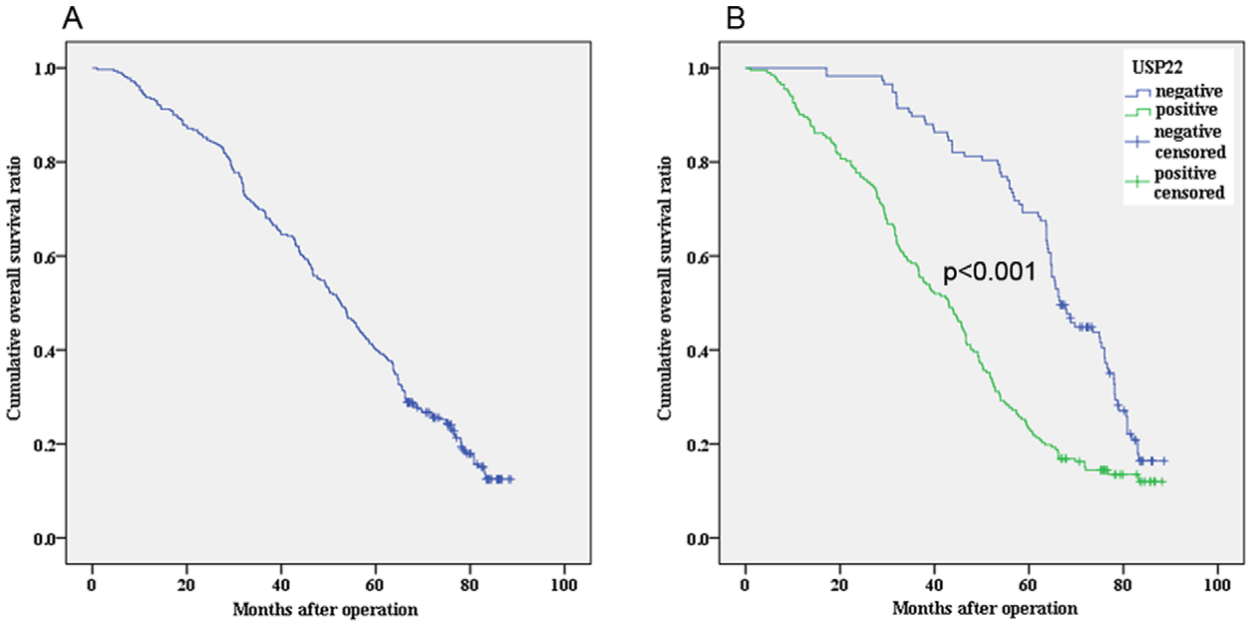
Comparison of overall survival according to USP22. A) Overall survival curves of all OSCC patients. B) Kaplan-Meier analysis revealed significantly poorer overall survival (*P*<0.001) of patients with positive expression of USP22.

**Figure 4:**
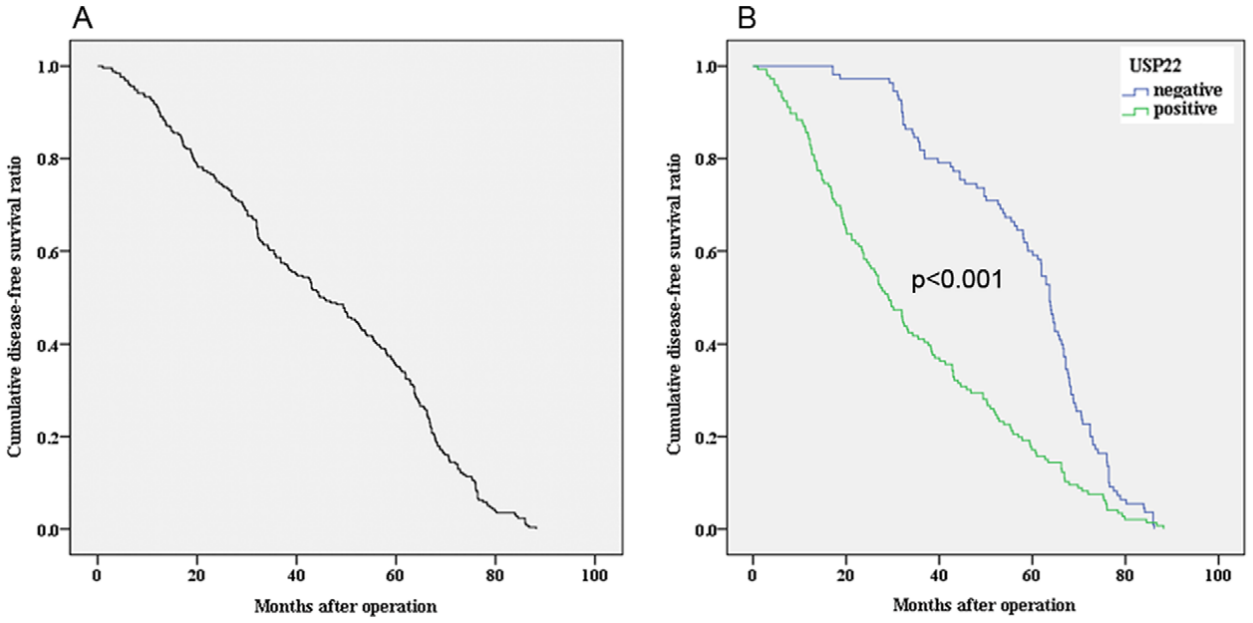
Comparison of disease-free survival according to USP22. A) Disease-free survival curves of all OSCC patients. B) Kaplan-Meier analysis revealed significantly poorer disease-free survival (*P*<0.001) of patients with positive expression of USP22.

We evaluated the prognostic value of USP22 on DFS and OS in all patients. The median OS for the all patients (n = 319) was 52.0±1.4 months. The 5 years of OS rate was 40.1%±2.7%. Kaplan–Meier analysis demonstrated that OSCC patients with positive expression of USP22 had a significantly poorer OS compared to the patients with negitive expression of USP22 (44.0±1.7 vs. 66.1±1.7 months, χ^2^ = 40.250, *P*<0.001) (Figure 3). The median DFS for the patients (n = 256) was 44.9±1.5 months. The 5 years of DFS rate was 35.5±3.0%. Kaplan–Meier analysis demonstrated that OSCC patients with positive expression of USP22 had a significantly poorer DFS compared to the patients with negitive expression of USP22 (34.6±1.9 vs. 58.7±1.6 months, χ^2^ = 37.475, *P*<0.001) (Figure 4). In addition, by Cox regression analysis, USP22 expression was recognized as an independent prognostic indicator of survival (Table 2). Therefore, our findings indicated that OSCC exhibiting nuclear USP22 expression are associated with increased metastatic potential and poor survival outcomes.

**Table 2:** Multivariate analyses of factors associated with OS and DFS.

Survival	Category	HR (95%)	P-value
**Overall survival**			
Tumor size	<3 vs. ≥3	1.285(0.999–1.652)	0.050
COX-2	Negative vs. positive	0.718(0.553–0.933)	0.013
USP22	Negative vs. positive	2.407(1.822–3.180)	<0.001
**Disease-free survival**			
M classification	M0 vs. M1	0.665(0.465–0.950)	0.025
COX-2	Negative vs. positive	0.707(0.544–0.920)	0.010
USP22	Negative vs. positive	2.743(2.056–3.659)	<0.001

### Influence of USP22 knockdown on Tca-8113 and Cal-27 cell proliferation and colony formation

**Figure 5:**
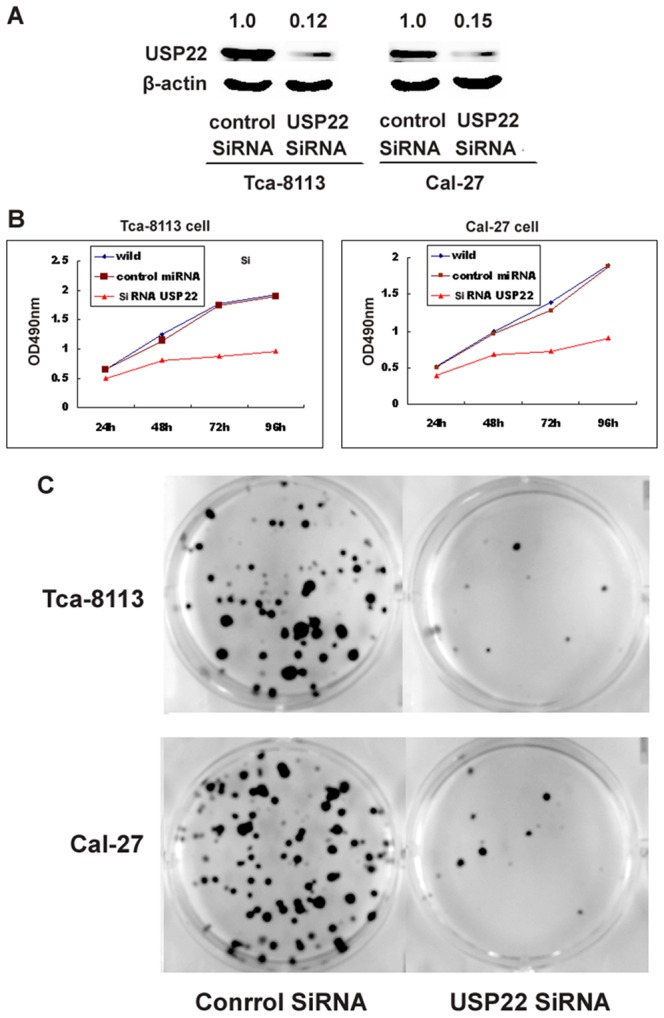
Knockdown of USP22 expression suppressed OSCC cell proliferation and colony formation. A) Two USP22 knockdown cell lines (Tca-8113 and Cal-27) and the corresponding control cells were established, as described in Materials and methods. The expression level of USP22 was determined using Western blot analysis. B) Compared with the control treated cells, the level of proliferation of OSCC cell transfected by USP22 siRNA significantly decreased by the MTT assay. C) In agreement with cell proliferation, USP22 siRNA decreased the number of colonies of the two cell lines.

To confirm USP22 function in the OSCC cell, USP22 expression was repressed by siRNA. Figure 5A showed that the expression of USP22 in USP22 siRNA-transfected cell lines were 0.12-fold and 0.15-fold, respectively, relative to that of the cells transfected with the control vector. To determine the effect of the knockdown of USP22 in OSCC cells, cell growth were assayed by MTT and colony formation assays. Figure 5B showed the viablility rates of the two USP22 knockdown cell lines and the two control cell lines. The two USP22 knockdown cell lines grew significantly (P<0.05) more slowly than did the the two control cell lines, suggesting that USP22 positively regulated cell proliferation. Figure 5C showed an image of the cell colonies. In agreement with cell proliferation, USP22 siRNA decreased the number of colonies of Tca-8113 and Cal-27 cells. These results suggested that USP22 siRNA effectively inhibited OSCC cell growth in vitro.

## Discussion

OSCC, a subgroup of head and neck cancers, represents more than 95% of all malignant neoplasia in the oral cavity [16]. Current treatment options are limited and overall 5-year survival is poor [17], [18]. The parameters based on clinical and histopathological variables often fail to predict the outcome of therapeutical options. Consequently, there is an urgent need to identify markers that can predict responses to therapy, inform individualized treatment strategies and provide insights into the mechanism of metastatic dissemination and therapy resistance.

11-gene Polycomb/cancer stem cell signature is used to effectively predict the therapeutic outcome of individual cancer patients [3], [19]. USP22, one gene of this signature, have been demonstrated to play a crucial role in pathological processes of epithelial malignancies and other solid tumors [4], [5], [7]. In the previous studies, we reported that USP22 may function as a highly promising diagnostic and/or prognostic marker [8]–[11]. However, no direct effects of USP22 to human OSCC progression were revealed. Based on this idea, the clinical significance of USP22 activation in OSCC was demonstrated in this study.

Pijnappel et al. propose that targeted deubiquitylation of histones is intimately linked to transcription activation, epigenetic regulation, and cancer progression [6]. USP22 edits the histone code by deubiquitinating H2A and H2B as part of the mammalian SAGA (Spt-Ada-Gcn5) complex [5], [7]. Thus, we proposed that USP22 activation may be implicated in the initiation and progression of cancer. We collected tissue samples representative of the OSCC spectrum including normal mucosa, carcinoma, and lymph node, to reveal whether alterations of USP22 expression may affect malignant transformation. We found that the USP22 expression increased significantly from normal mucosa to carcinomas and from carcinomas to lymph node metastasis. These findings strongly suggested that USP22 activation may play an oncogenic role in promoting tumor progression and metastatic dissemination to lymph node.

To further confirm our hypothesis, we analyzed the clinical significance of USP22 expression in OSCC. Among the 319 OSCC samples, the positive frequency was 63.32% for USP22. As to clinicopathological variables, there was a significant difference of USP22 expression in patients categorized according to primary tumor site, tumor size, differentiation, T classification, N classification, M classification, AJCC stage, Ki-67 status, and COX-2 status, which strongly suggesting that USP22 can be used as a marker to classify subsets of OSCC patients with more aggressive disease. Moreover, the prognostic power of USP22 was examined in the patients with OSCC. Cox regression analysis revealed that USP22 expression was recognized as an independent prognostic indicator of survival. Kaplan–Meier analysis confirmed that OSCC patients with USP22 high expression had a significantly poorer OS and DFS compared to the patients with USP22 low expression. Those results represented that USP22 expression was associated with increased propensity to develop metastasis, high likelihood of treatment failure, and increased probability of death from cancer after surgery. Moreover, we also observed knockdown of USP22 expression reduced OSCC cell proliferation and colony formation, which made the further evidence about the involvement of USP22 in oncogenesis and metastasis of OSCC. In fact, our results was consisted with previous studies which revealed that the 11-gene Polycomb/cancer stem cell signature, reveals a genetic link between the stemness state and clinically lethal death-from-cancer phenotypes, which seems highly informative in stratification into subgroups with therapy failure [3], [5]. Thus, our results supported this hypothesis that cancer cells expressing USP22 may manifest a stem cell-like characteristics such as therapy-resistant and metastasis-enabling phenotypes [3].

The precise mechanisms by which USP22 expression affects cancer progression and metastasis is unclear. Only few papers analyze the possible mechanism of USP22. Atanassov et al report that USP22 regulates cell proliferation and tumorigenesis by deubiquitinating the transcriptional regulator FBP1 [20]. Chipumuro et al demonstrate that the ubiquitin hydrolase USP22 contributes to 3′-end processing of JAK-STAT-inducible genes [21]. We previously showed that USP22 acted as an oncogene by the activation of BMI-1-mediated INK4a/ARF pathway and Akt pathway [13]. However, how do USP22 control these signatures? Combine with the previous studies, we proposed that histone modification played the key function in regulating the gene activation. USP22, a subunit of the hSAGA complex, is identified to remove Ub efficiently both from histone H2A and H2B. The ubiquitination/deubiquitination cycle of histones H2A and H2B is important in regulating chromatin dynamics and transcription mediated, in part, via “cross-talk” between histone ubiquitination and methylation. For example, H2B ubiquitination is a pre-requisite for di- and tri-methylation of Lys4 and Lys79 of histone H3 (H3K4me and H3K79me), a modification that correlates with transcriptionally active chromatin and helps recruit the SAGA co-activator complex and additional chromatin modification activities [22]–[24]. However, many unanswered questions remain about this modification and its role in chromatin and transcription. Further mechanistic studies will be required to address the exact link between these activities and the other chromatin-modifying complexes to understand how these sequential events participate in chromatin remodeling and gene activation.

In summary, we reported that USP22 activated in a majority of clinical samples of OSCC and increased of USP22 expression exhibited a marked propensity toward highly malignant clinical behavior, lymph node metastasis, and poor survival after surgery. Thus, the identification of USP22 in OSCC can potentially serve as a new prognostic indicator predicting the treatment outcome of OSCC.
